# Choline metabolism reprogramming mediates an immunosuppressive microenvironment in non-small cell lung cancer (NSCLC) by promoting tumor-associated macrophage functional polarization and endothelial cell proliferation

**DOI:** 10.1186/s12967-024-05242-3

**Published:** 2024-05-10

**Authors:** Bijing Xiao, Guanjun Li, Haimiti Gulizeba, Hong Liu, Xiaoxian Sima, Ting Zhou, Yan Huang

**Affiliations:** 1 Medical Oncology Department, State Key Laboratory of Oncology in South China, Sun Yat-Sen University Cancer Center, Collaborative Innovation Center for Cancer Medicine, No. 651 Dongfeng East Road, Guangzhou, 510060 Guangdong People’s Republic of China; 2 Department of Oncology, Nanfang Hospital, Southern Medical University, No. 1023-1063, Shatai Southern Road, Baiyun District, Guangzhou, 510060 Guangdong People’s Republic of China

**Keywords:** Non-small cell lung cancer, Cholinesterase, Choline metabolism, Immunotherapy response, Metabolic reprogramming, Immune microenvironment, Macrophage, Endothelial cell proliferation

## Abstract

**Introduction:**

Lung cancer is a prevalent malignancy globally, and immunotherapy has revolutionized its treatment. However, resistance to immunotherapy remains a challenge. Abnormal cholinesterase (ChE) activity and choline metabolism are associated with tumor oncogenesis, progression, and poor prognosis in multiple cancers. Yet, the precise mechanism underlying the relationship between ChE, choline metabolism and tumor immune microenvironment in lung cancer, and the response and resistance of immunotherapy still unclear.

**Methods:**

Firstly, 277 advanced non-small cell lung cancer (NSCLC) patients receiving first-line immunotherapy in Sun Yat-sen University Cancer Center were enrolled in the study. Pretreatment and the alteration of ChE after 2 courses of immunotherapy and survival outcomes were collected. Kaplan–Meier survival and cox regression analysis were performed, and nomogram was conducted to identify the prognostic and predicted values. Secondly, choline metabolism-related genes were screened using Cox regression, and a prognostic model was constructed. Functional enrichment analysis and immune microenvironment analysis were also conducted. Lastly, to gain further insights into potential mechanisms, single-cell analysis was performed.

**Results:**

Firstly, baseline high level ChE and the elevation of ChE after immunotherapy were significantly associated with better survival outcomes for advanced NSCLC. Constructed nomogram based on the significant variables from the multivariate Cox analysis performed well in discrimination and calibration. Secondly, 4 choline metabolism-related genes (MTHFD1, PDGFB, PIK3R3, CHKB) were screened and developed a risk signature that was found to be related to a poorer prognosis. Further analysis revealed that the choline metabolism-related genes signature was associated with immunosuppressive tumor microenvironment, immune escape and metabolic reprogramming. scRNA-seq showed that MTHFD1 was specifically distributed in tumor-associated macrophages (TAMs), mediating the differentiation and immunosuppressive functions of macrophages, which may potentially impact endothelial cell proliferation and tumor angiogenesis.

**Conclusion:**

Our study highlights the discovery of ChE as a prognostic marker in advanced NSCLC, suggesting its potential for identifying patients who may benefit from immunotherapy. Additionally, we developed a prognostic signature based on choline metabolism-related genes, revealing the correlation with the immunosuppressive microenvironment and uncovering the role of MTHFD1 in macrophage differentiation and endothelial cell proliferation, providing insights into the intricate workings of choline metabolism in NSCLC pathogenesis.

**Supplementary Information:**

The online version contains supplementary material available at 10.1186/s12967-024-05242-3.

## Introduction

Lung cancer is one of the most prevalent malignancies worldwide and still has the highest rate of cancer-related mortality, with non-small cell lung cancer (NSCLC) being the most common type in which lung adenocarcinoma (LUAD) is the most common histologic type [1]. Over the past decades, immunotherapy has revolutionized the treatment landscape of NSCLC and has emerged as a standard first-line therapy for advanced or metastatic NSCLC patients with negative driver-gene mutation [2, 3]. However, there remains a substantial proportion of patients did not benefit from immunotherapy and some patients present primary or secondary resistance, contributing to poor therapeutic effect and dismal prognosis. Therefore, there is a pressing need to identify accurate and efficient biomarkers and to explore the mechanisms of resistance to immunotherapy.

Cholinesterase (ChE) is a glycoprotein synthesized by the liver and secreted into the blood, which are involved in a diverse range of physiological and pathological processes, including cellular growth, differentiation, apoptosis, inflammation and cell metabolism [4, 5]. Moreover, accumulating evidence showed that the decreased level or activity of ChE may play an important role in the development and progression of tumors and may be associated with the poor prognosis of multiple cancers, including lung cancer [6], gastric cancer [7], prostate cancer [8] and cervical cancer [9]. However, to our best known, no studies have yet reported the protective role of ChE in tumor immunity.

Moreover, ChE is intimately tied to choline metabolism. ChE is a key enzyme responsible for catalyzing the hydrolysis of acetylcholine (ACh) into choline and acetic acid [10], playing a crucial role in maintaining a balanced choline metabolism. Choline metabolism closely involves in protecting the integrity of cell membrane, coordinating the methylation and synthesizing important neurotransmitters [11]. In cancer, the rapid proliferation of malignant tumor cells promotes a large amount of choline uptake through the overexpression of enzymes, variations in choline transporters and changes in signaling pathways, which result in the dysregulation of choline metabolism [12]. Abnormal choline metabolism has emerged as a metabolic hallmark of tumor oncogenesis and progression [13]. Recent research has indicated that choline metabolism-related signature is associated with the immune microenvironment of colon adenocarcinoma patients and has potential application value in predicting the prognosis and chemotherapy response [14]. Specifically, they found that patients in the high-risk group of choline metabolism had an elevated levels of CD8 + T cells and Treg cells. The presence of immunosuppressive factors in the tumor microenvironment likely hinders the antitumor function of CD8 + T cells, contributing to the poorer prognosis observed in the high-risk group of choline metabolism and potentially leading to worse efficacy of immunotherapy. Nevertheless, the precise mechanism of the relationship between choline metabolism and tumor immune microenvironment in NSCLC, and the response and resistance of immunotherapy still need to be elucidated.

In this study, we assess the effect and potential predictive and prognostic value of ChE NSCLC patients undergoing immunotherapy. Meanwhile, a risk model based on choline metabolism-related genes was developed, and single-cell analysis was used to explore the interconnection between choline metabolism and tumor microenvironment. Our findings suggest that ChE may be effective to predict prognosis and immunotherapy efficacy of NSCLC and provide an important reference value for treatment. Furthermore, our study indicates that methylenetetrahydrofolate dehydrogenase 1 (MTHFD1), a key suppressor gene in choline metabolism, is correlated with an immunosuppressive microenvironment and involved in macrophage differentiation as well as endothelial cell proliferation, thus contributing to a deeper understanding of the intricate mechanisms underlying choline metabolism in the development of NSCLC.

## Materials and methods

### Patient selection

298 advanced and treatment-naïve NSCLC patients treated with immunotherapy at Sun Yat-sen University Cancer Center between August 2018 and April 2023 were screened in this retrospective, observational study. Exclusion criteria included: receiving prior anticancer therapy; lacking baseline or the alteration level of ChE after 2 cycles of immunotherapy; lacking efficacy and survival data; loss to follow-up. 21 patients who met the exclusion criteria were excluded and finally 277 eligible patients were enrolled in our study. The flow chart of patients screening was summarized in Additional file 1: Figure S1. Ethics approval for this study, including a waiver of informed consent, was achieved from Sun Yat-sen University Cancer Center Institutional Review Board (SL-B2022-680-02).

### Clinical data collection and outcomes

Patients baseline characteristics including age, gender, Eastern Cooperative Oncology Group performance status (ECOG-PS), smoking status, distant metastasis and treatment regimens were documented. In addition, baseline laboratory parameters (within 7 days prior to the treatment initiation) including cholinesterase, lactate dehydrogenase (LDH), serum albumin (ALB), C-reactive protein (CRP), serum amyloid A (SAA) and neutrophil–lymphocyte ratio (NLR = absolute neutrophil count/absolute lymphocyte count) were collected and analyzed. Meanwhile, ChE level after 8 [± 2] weeks after the initiation of immunotherapy was also collected and all the patients were then categorized into ChE-increased group and ChE-decreased group based on the alteration level of ChE.

Tumor response and progression were regularly performed according to Response Evaluation Criteria in Solid Tumors version 1.1 (RECIST 1.1) per Response Evaluation Criteria. The primary endpoint was overall survival (OS) defined as the time from initiation of immunotherapy to death from any causes. The secondary endpoint was progression-free survival (PFS) calculated from initiation of immunotherapy to tumor-progression or death from any causes. Patients who had not progressed or are not deceased were censored at the time of the last follow up.

### Establishment of a prognostic nomogram

Those significant clinical predictors observed in multivariate Cox model (*p* < 0.05) were then used to construct a nomogram. Then, we calculated the sum points of each patient based on the constructed nomogram and the median level of total points were used to stratified patients into high/low-point groups. Time-dependent receiver operating characteristic (ROC) curves at 1-, 2-, 3-year OS were plotted, and the area under the curve (AUC) was calculated to evaluate the predictive value of the nomogram. The prediction probability and the observed result frequency were compared through the calibration curve.

### Genomic data collection

RNA sequencing (RNA-seq) data of 600 LUAD samples, including 513 cancer samples and 87 adjacent normal samples were downloaded from The Cancer Genome Atlas (TCGA) database (https://portal.gdc.cancer.gov/projects/TCGA-LUAD). The clinical information including age, gender, TNM stage, survival time and survival status were also obtained.

In addition, the ORIENT-11 study, served as a validation dataset, is a multi-center, randomized, double-blind, phase 3 study which enrolled 398 advanced or metastatic NSCLC patients in China [15], receiving sintilimab (anti-PD1) plus pemetrexed and platinum (experiment group) or placebo plus pemetrexed and platinum (control group). 227 patients lacking RNA-seq data and 58 patients in control group (did not receive anti-PD1 therapy) were excluded in our study. Finally, the RNA-seq data as well as the clinical information of the 113 patients from ORIENT-11 study was collected for further analysis.

### Construction and validation of a choline metabolism risk model

Choline metabolism-related genes were obtained from the Kyoto Encyclopedia of Genes and Genomes (KEGG) database, AmiGO2 website and Reactome Pathway Databases (Additional file 2: Table S1). Univariate and multivariate Cox analysis were performed to identify significant and independent OS-associated choline metabolism-related genes in TCGA-LUAD database. The risk scores of each patient both in TCGA database and the orient-11 study (the validation cohort) calculated using the following formula.

$${\text{Risk}}\,{\text{score}}\, = \,\mathop \sum \limits_{{{\text{n}} = 1}}^{{\text{n}}} ({\text{coef}}_{{\text{i}}} \, \times \,{\text{x}}_{{\text{i}}} )$$

Patients were then classified into high-risk and low-risk group based on the median level of risk score. R package of “survminer” were used to plot the risk curves and survival curves of two risk groups and “survivalROC” package were conducted to draw the ROC curves.

### Functional enrichment and immune microenvironment analysis

The "limma" R package was utilized to identify differentially expressed genes (DEGs) between the high-risk and low-risk groups in TCGA-LUAD. Using the R package “clusterProfiler” [16], GO function enrichment analysis and KEGG pathway enrichment analysis of the DEGs were performed. Additionally, the "msigdbr" R package was used to perform Gene Set Enrichment Analysis (GSEA) on the DEGs to analyze the signaling pathways enrichment in different risk-groups. "IOBR" R package [17] was used to calculate the immune infiltration score and metabolism score for high-risk and low-risk patients. The Tumor Immune Dysfunction and Exclusion (TIDE) score [18], a computational method to model two primary mechanisms of tumor immune evasion, were downloaded from the TIDE database (http://tide.dfci.harvard.edu/) after following the instructions on the website to uploaded input data, and the TIDE scores in two risk groups were analyzed. Besides, the scoring data of immune checkpoint inhibitors (ICIs) treatment were downloaded from the Cancer Immunome Database (TCIA) (https://tcia.at/) [19].

### Single-cell sequencing data collection and processing

The single-cell dataset GSE207422 of resectable non-small cell lung cancer (NSCLC) before and after PD-1 blockade combined with chemotherapy was downloaded from Gene Expression Omnibus (GEO) database, and quality control was performed on the raw gene expression matrix using the Seurat R package. The following criteria were applied for filtering cells: exclusion of cells with less than 200 or more than 6000 expressed genes, those with the proportion of mitochondrial gene expression in UMI count was more than 20%, and filtering out genes expressed in fewer than 3 cells. After performing quality control on the raw gene expression matrix, we processed and analyzed the data using the following steps: Firstly, the transcript counts were log-transformed using the NormalizeData function. Subsequently, the FindVariableFeatures function was employed to select the top 2,000 genes with the highest variability within cells. Next, the ScaleData function was applied to normalize the gene expression data, ensuring comparability among genes. To reduce the dimensionality of the data and capture the major sources of variation, we performed linear Principal Component Analysis (PCA) using the RunPCA function from the Seurat package. To correct for batch effects, we employed the Harmony algorithm [20] based on the samples. For clustering analysis, we chose a clustering resolution of 0.6 to better distinguish distinct cell populations. Visualization of cell clusters was performed using the Uniform Manifold Approximation and Projection (UMAP) dimensionality reduction technique. Finally, all cells were annotated based on the expression patterns of cell-specific marker genes.

### Cell function scores

To define the functional states of myeloid cell clusters, the Ucell was used to calculate functional scores for each cluster based on known functional genes specific to myeloid cells.

### Single-cell trajectory inference

Monocle 3 [21] was applied to infer the cell differentiation trajectories of myeloid cell clusters. The functions "cluster_cells" and "learn_graph" were used to partition the myeloid cells and fit the principal graph, while the UMAP visualization was employed to visualize the differentiation trajectories. We selected CD14^+^ monocytes as the starting point to infer the cell differentiation trajectories of myeloid cells.

### Cell–cell interaction analysis

For the analysis of cell–cell interactions, the CellChat [22] was utilized to predict the communication patterns of cell–cell receptors and ligands from the single-cell transcriptomic data. Special attention was given to the communication strength between myeloid cells and other cells in the tumor microenvironment.

## Results

### Patient characteristics at baseline

A total of 277 treatment-naïve patients with advanced non-small cell lung cancer, who were treated with first-line anti-PD1 therapy in Sun Yat-sen University Cancer Center between August 3, 2018, and April 19, 2023 were enrolled in the study. Patients ranged in age from 32 to 84 years old, with a median age of 62. Among all the included patients, 222 (80.1%) were males, 135 (48.7%) had an ECOG PS of 0, 183 (66.1%) were current or former smoker, 38 (13.7%) had liver metastasis and 95 (34.3%) had brain metastasis at baseline. In total, 233 (84.1%) patients received chemotherapy plus immunotherapy, 38 (13.7%) received chemotherapy plus immunotherapy combined with antiangiogenic therapy, 3 (1.1%) received single-agent immunotherapy, and the other 3 received immunotherapy combined with antiangiogenic therapy. The baseline clinical characteristics of the patients were summarized in Table 1. Based on the median value of baseline ChE (7611 units/liter, U/L), all the patients were the stratified into two groups. Clinicopathological baseline characteristics were comparable between the two groups (Table 2). Results showed that age, smoking status, high ALB, low NLR, low CRP and low SAA were significantly associated with high baseline ChE level (*p* < 0.05). At the last follow-up (December 4, 2023), 123 (44.4%) patients died, the median progression-free survival time was 10.44 months (95% CI 8.37–12.50 months) and the median overall survival was 34.17 months (95% CI 30.47–37.86 months).

**Table 1:** Patients characteristics at baseline (n = 277)

Characteristics	No. of patients (%)
Age, years	
Median(range)	62 (32–84)
< 62	139 (50.2)
≥ 62	138 (49.8)
Gender	
Male	222 (80.1)
Female	55 (19.9)
ECOG-PS	
0	135 (48.7)
1–2	142 (51.3)
Smoking status	
Never smoker	94 (33.9)
Current or former smoker	183 (66.1)
Metastases (baseline)	
Liver	38 (13.7)
Brain	95 (34.3)
Bone	103 (37.2)
Treatment type	
Single-agent immunotherapy	3 (1.1)
Immunotherapy + chemotherapy	233 (84.1)
Immunotherapy + antiangiogenic therapy	3 (1.1)
Immunotherapy + chemotherapy + antiangiogenic therapy	38 (13.7)
Baseline ChE, U/L	
< 7611	138 (49.8)
≥ 7611	139 (50.2)
ChE alteration	
Reduction	92 (33.2)
Elevation	185 (66.8)

Data are expressed as the median or number (%)

*n* number of patients, *ECOG-PS* Eastern Cooperative Oncology Group performance status, *ChE* Cholinesterase

**Table 2:** Comparison of clinicopathological characteristics based on baseline ChE strata(n = 277)

Characteristics	ChE < 7611 (n = 139) *n* (%)	ChE ≥ 7611 (n = 138) *n* (%)	*p* value^Φ^
Age, years			**0.010**
Median(range)	62 (32–84)	61 (31–77)	
< 62	81 (58.3)	58 (42.0)	
≥ 62	58 (41.7)	80 (58.0)	
Gender			0.066
Male	118 (84.9)	104 (75.4)	
Female	21 (15.1)	34 (24.6)	
ECOG-PS			0.765
0	66 (47.5)	69 (50.0)	
1–2	73 (52.5)	69 (50.0)	
Smoking status			**0.007**
Never smoker	36 (25.9)	58 (42.0)	
Current or former smoker	103 (74.1)	80 (58.0)	
Metastases (baseline)			
Liver	24 (17.3)	14 (10.1)	0.122
Brain	47 (33.8)	48 (34.8)	0.965
Bone	55 (39.6)	48 (34.8)	0.484
LDH, U/L			0.133
< 210	63 (45.3)	76 (55.1)	
≥ 210	76 (54.7)	62 (44.9)	
ALB, g/L			** < 0.001**
< 42	99 (71.2)	40 (29.0)	
≥ 42	40 (28.8)	98 (71.0)	
NLR			**0.005**
< 3.18	57 (41.0)	81 (58.7)	
≥ 3.18	82 (59.0)	57 (41.3)	
CRP, mg/L			** < 0.001**
< 11	47 (33.8)	93 (67.4)	
≥ 11	92 (66.2)	45 (32.6)	
SAA, mg/L			** < 0.001**
< 20	49 (35.3)	90 (65.2)	
≥ 20	90 (64.7)	48 (34.8)	

Data are expressed as the median or number (%)

*n* number of patients, *ECOG-PS* Eastern Cooperative Oncology Group performance status, *ChE* Cholinesterase, *LDH* lactate dehydrogenase, *ALB* serum albumin, *NLR* neutrophil-to-lymphocyte ratio, *CRP* C-reactive protein, *SAA* serum amyloid A

^Φ^p values in boldface indicate p < 0.05

### The prognostic values of baseline and early changes in ChE for survival

**Fig. 1:**
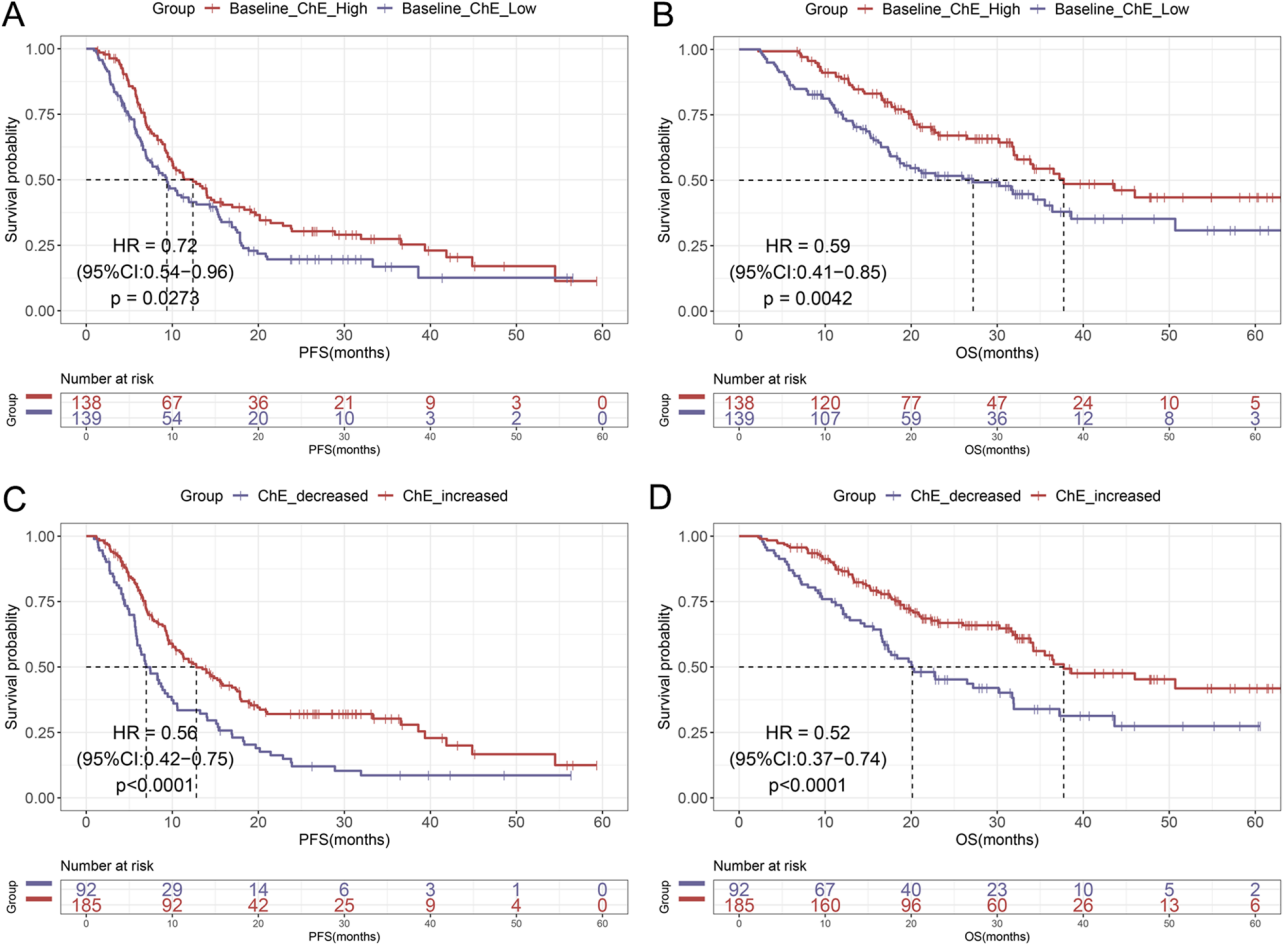
Kaplan–Meier curves of PFS (**A**) and OS (**B**) in patients with low/high ChE at baseline. Kaplan–Meier curves of PFS (**C**) and OS (**D**) in patients with decreased/increased levels of ChE after immunotherapy

According to ChE levels at baseline (with the median value as the cut-off), all the patients were divided into two groups (low group: ChE < 7611 U/L; high group: ChE ≥ 7611 U/L). Based on the alteration levels of ChE after the treatment of immunotherapy, patients were also stratified into ChE reduction group and ChE elevation group. First, we explored a prognostic role for baseline ChE levels. Kaplan–Meier survival analysis and log-rank test showed that patients with high ChE at baseline had both significantly prolonged PFS [median PFS, 12.40 vs. 9.37 months; HR = 0.72, (95% CI 0.54–0.96), *p* = 0.027; Fig. 1A] and OS [median OS, 37.73 vs. 27.20 months; HR = 0.59, (95% CI 0.41–0.85), *p* = 0.004; Fig. 1B] than those patients with low ChE at baseline. Second, the prognostic value of alteration levels of ChE was evaluated. We noted the similar trend that patients with an elevation of ChE had better PFS [median PFS, 12.80 vs. 6.97 months; HR = 0.56, (95% CI 0.42–0.75), *p* < 0.0001; Fig. 1C] and OS [median OS, 37.73 vs. 20.13 months; HR = 0.52, (95% CI 0.37–0.74), *p* < 0.0001; Fig. 1D] than those with a reduction of ChE.

**Fig. 2:**
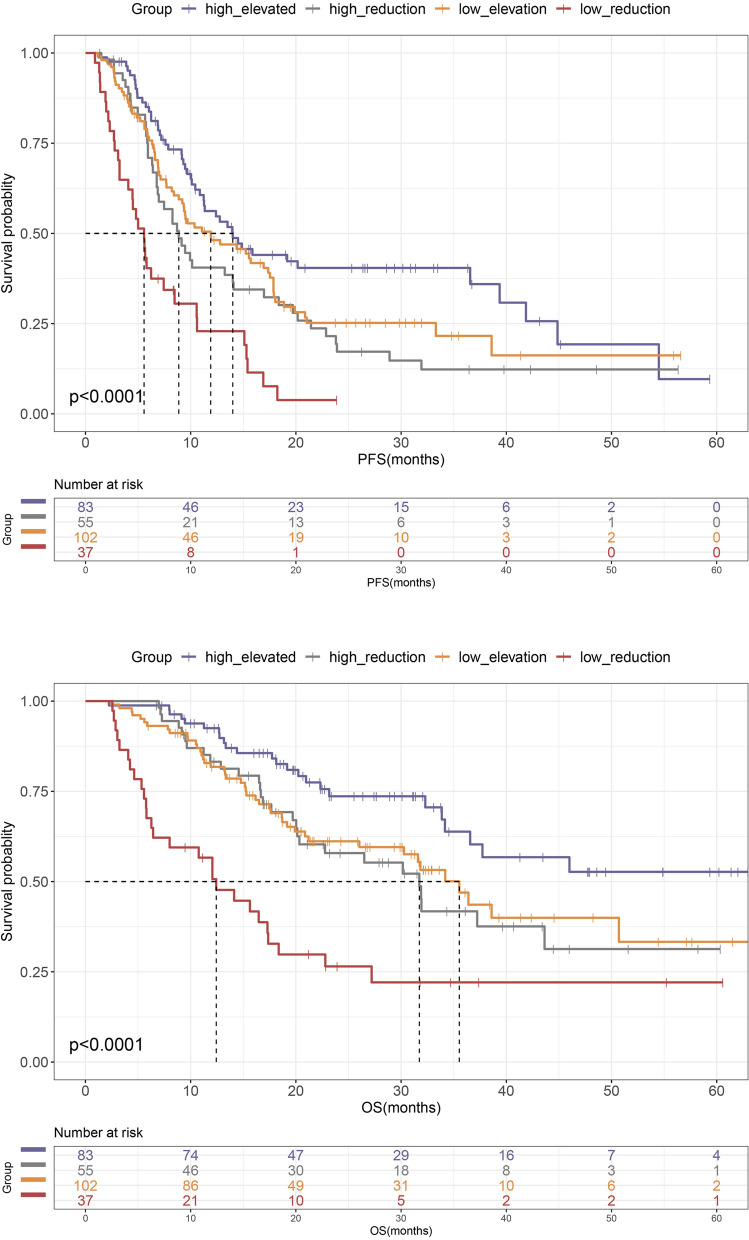
Kaplan–Meier curves of PFS (**A**) and OS (**B**) according to the combination of baseline ChE and the alteration of ChE after immunotherapy

For additional verification, patients were stratified into four groups by both baseline and the alteration of ChE after immunotherapy (baseline ChE low/high and the alteration of ChE reduction/elevation). Results indicated that the combination of both two prognostic factors improved risk stratification and prognostication for survival outcomes in advanced NSCLC (*p* < 0.0001) (Fig. 2). As expected, those with both high ChE at baseline as well as an elevation of ChE had the best survival outcomes of benefit (n = 83, median PFS = 14.00 months, median OS not reached). In addition, patients with either adverse prognostic feature (baseline low ChE or early reduction of ChE) had intermediate survival. While, Patients who had the low ChE at baseline and an early reduction of ChE after immunotherapy (n = 37) had the particular high risk of death and the worst survival outcomes, with a median PFS of 5.56 months and a median OS of 12.43 months (Fig. 2A, B). Therefore, both baseline high ChE and an elevation of ChE were independently displayed a better clinical benefit from immunotherapy, and the combination of these two prognostic factors can further improve risk stratification.

### Univariate and multivariate cox regression analyses of PFS and OS

Based on the univariate analysis, liver metastasis (*p* < 0.001), bone metastasis (*p* = 0.006), baseline ChE (*p* = 0.027) and the alteration of ChE (*p* < 0.001) were significantly associated with PFS (Additional file 2: Table S2). The multivariate Cox regression analysis revealed that liver metastasis (*p* = 0.001), bone metastasis (*p* = 0.009), baseline ChE (*p* = 0.004) and the alteration of ChE (*p* < 0.001) were the independent indicators of PFS. Furthermore, univariate and multivariate Cox regression analyses were also performed for identifying OS prognostic factors. Univariate analysis revealed that factors associated with inferior OS included male, age ≥ 62 years old, current or former smoker, liver metastasis, bone metastasis, low ChE at baseline (*p* = 0.004), a reduction level of ChE (*p* < 0.001), low ALB, high CRP and high SAA. We further included those significant clinicopathological parameters in univariate analysis into the multivariate model. Multivariate analysis revealed that baseline ChE (high vs. low, HR, 0.65; 95% CI 0.43–0.99; *p* = 0.047) and ChE alteration (elevation vs. reduction, HR, 0.51; 95% CI 0.35–0.74; *p* < 0.001) were independent indicators for OS, others including age (< 62 vs. ≥ 62 years old, HR, 0.67; 95% CI 0.46–0.98; *p* = 0.037), liver metastasis (Yes vs. No, HR, 1.73; 95% CI 1.09–2.75; *p* = 0.019) and bone metastasis (Yes vs. No, HR, 2.23; 95% CI 1.55–3.22; *p* < 0.001) at baseline (Table 3).

**Table 3:** Predictive factors for OS by univariate and multivariate analysis

		Univariate analyses		Multivariate analyses	
		HR (95%CI)	*p* value^Φ^	HR (95%CI)	*p* value^Φ^
Gender	Male vs. Female	1.92 (1.15–3.21)	**0.013**	1.39 (0.72–2.67)	0.324
Age	< 62 vs. ≥ 62	0.55 (0.38–0.79)	**0.001**	0.67 (0.46–0.98)	**0.037**
ECOG-PS	1–2 vs. 0	1.21 (0.85–1.72)	0.298		
Smoking status	Yes vs. No	1.64 (1.10–2.42)	**0.014**	1.03 (0.63–1.70)	0.901
Liver metastasis	Yes vs. No	2.04 (1.30–3.19)	**0.002**	1.73 (1.09–2.75)	**0.019**
Brain metastasis	Yes vs. No	0.88 (0.59–1.30)	0.511		
Bone metastasis	Yes vs. No	2.29 (1.60–3.27)	** < 0.001**	2.23 (1.55–3.22)	** < 0.001**
Baseline ChE, U/L	≥ 7611 vs. < 7611	0.59 (0.41–0.85)	**0.004**	0.65 (0.43–0.99)	**0.047**
ChE alteration, U/L	≥ 0 vs. < 0	0.52 (0.37–0.74)	** < 0.001**	0.51 (0.35–0.74)	** < 0.001**
LDH, U/L	≥ 210 vs. < 210	0.99 (0.70–1.41)	0.967		
ALB, g/L	≥ 42 vs. < 42	0.65 (0.46–0.93)	**0.018**	0.97 (0.61–1.55)	0.897
NLR	≥ 3.18 vs. < 3.18	1.21 (0.85–1.73)	0.284		
CRP, mg/L	≥ 11 vs. < 11	1.86 (1.30–2.67)	**0.001**	1.39 (0.72–2.69)	0.321
SAA, mg/L	≥ 20 vs. < 20	1.70 (1.19–2.44)	**0.004**	1.04 (0.55–1.97)	0.907

*OS* overall survival, *ECOG-PS* Eastern Cooperative Oncology Group performance status, *ChE* Cholinesterase, *LDH* lactate dehydrogenase, *ALB* serum albumin, *NLR* neutrophil-to-lymphocyte ratio, *CRP* C-reactive protein, *SAA* serum amyloid A, *HR* Hazard ratio, *CI* Confidence interval

^**Φ**^Values in boldface indicate *p* < 0.05

### Prognostic nomogram for prediction of OS

**Fig. 3:**
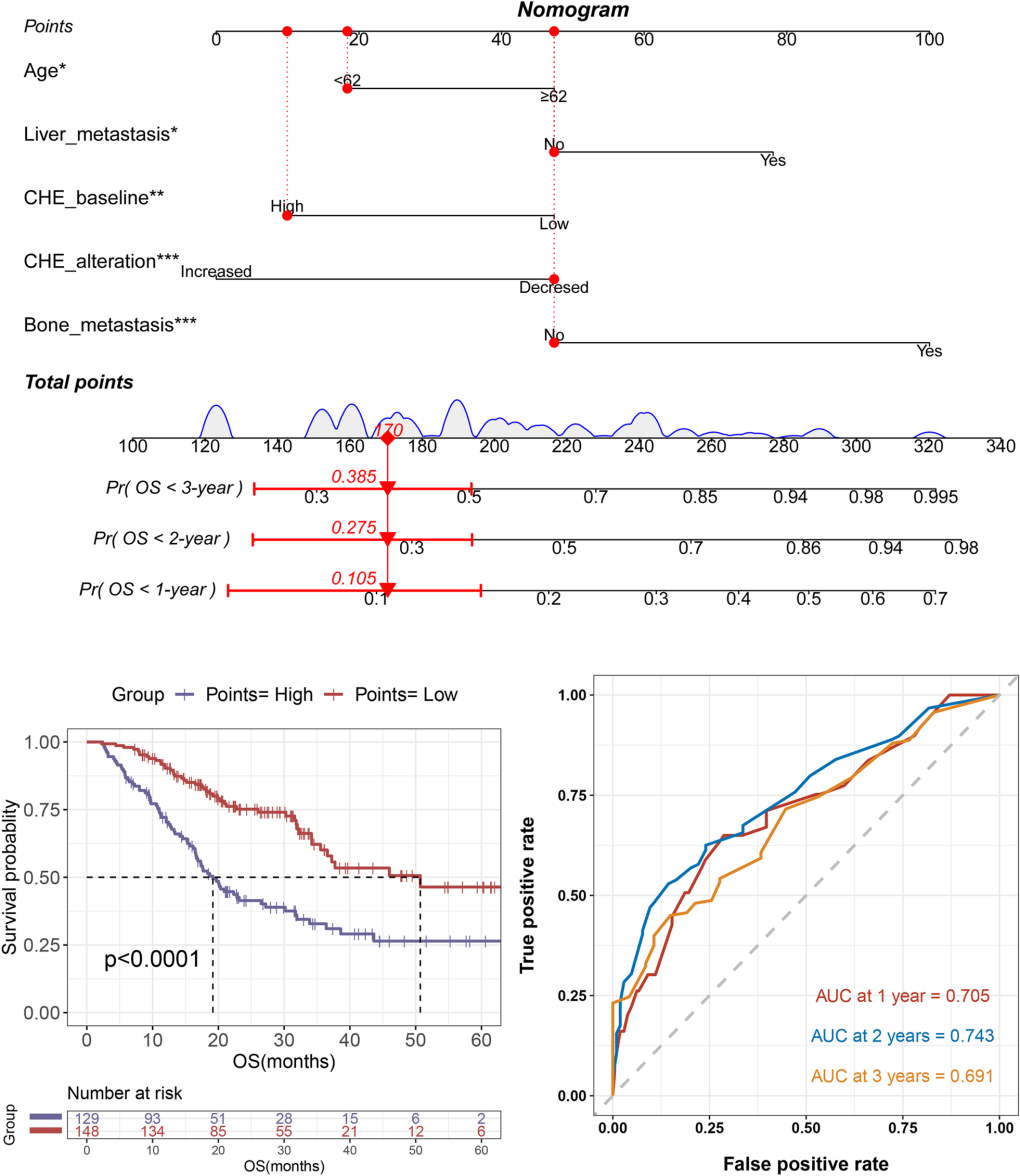
Construction of a prognostic nomogram. **A** Prognostic nomogram for predicting the probability of 1-, 2- and 3-year OS in NSCLC patients after immunotherapy. **B** Time-dependent ROC curves and AUCs at 1-, 2-, and 3- year- were plotted to verify the prognostic accuracy of the nomogram. **C** Kaplan–Meier curves for OS with risk stratification

The significant variables from the multivariate Cox analysis, including age, liver metastasis, bone metastasis, baseline ChE and alteration of ChE were used to establish a prognostic nomogram for OS. Each variable corresponds to a specific point by drawing a line straight up to the score axis, and the total points were calculated by adding up the individual score of each of the 5 variables included in the nomogram. The probability of survival was demonstrated by making a vertical line from the total score axis to intersect the survival probability axis of 1, 2, and 3 years (Fig. 3A). Prognostic accuracy and predictive value of nomogram were evaluated using the time-dependent ROC curves and AUC. The AUC values in the ROC curve analysis showed a good accuracy, with the AUC values of 1-, 2-, and 3-year survival probability of 0.705, 0.743 and 0.691 (Fig. 3B). The calibration plots demonstrated satisfactory consistency between the nomogram-predicted OS and actual observed probability of OS at 1-, 2- and 3-year (Additional file 3: Fig. S2A).

Based on the constructed nomogram, we calculated the total risk score for the enrolled NSCLC patients, and all of them were then divided into high-risk and low-risk groups, according to the median value of total point. As showed in the Kaplan Meir curve, a significant difference in OS were observed that patients in the high-risk group had remarkably worse survival outcomes than patients in the low-risk group (*p* < 0.0001) (Fig. 3C). Finally, the plotted decision-curve analysis (DCA) curve demonstrated the clinical validity and usefulness of the model (Additional file 3: Fig.S2B).

### Construction and verification of choline metabolism-related genes signature

**Fig. 4:**
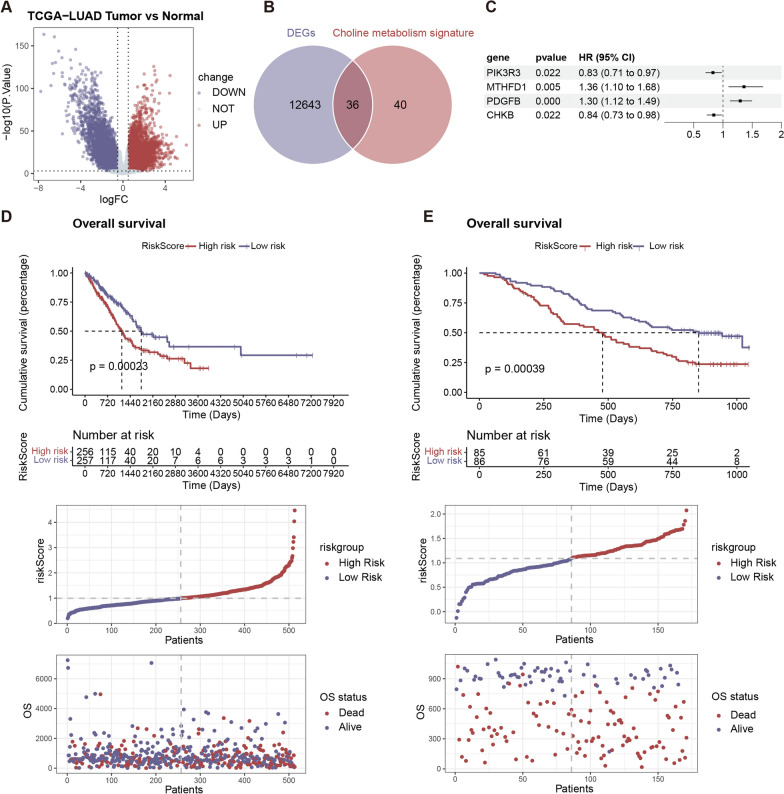
Choline metabolism signature based on RNA-seq analysis. **A** Identification of DEGs between tumor and normal tissues in TCGA-LUAD through transcriptomic sequencing data analysis and functional enrichment analysis. **B** Venn diagram demonstrating the intersections of DEGs and choline metabolism-related genes. **C** Forest plot of multivariate Cox regression analysis. **D**–**E** Kaplan–Meier survival curves, risk scores and survival status of the low- and high-risk groups of choline metabolism in training cohort (**D**) and validation cohort (**E**)

To further explore the gene changes and specific mechanisms related to choline metabolism, we obtained bulk RNA-seq data and clinical information of LUAD patients from TCGA database (https://cancergenome.nih.gov/). “Limma” package in R was used to identify DEGs between LUAD and normal tissues (Fig. 4A). The Venn diagrams were plotted to show the overlap between DEGs in LUAD and choline metabolism-related genes obtained from KEGG, Reactome Path and AmiGO2 databases (Fig. 4B). We finally screened 36 choline metabolism-related DEGs, including 14 upregulated genes and 22 downregulated genes. Subsequently, univariate and multivariate Cox regression analysis of the choline metabolism-related DEGs were performed and we finally obtain four choline metabolism-related prognostic genes (MTHFD1, PDGFB, PIK3R3, CHKB) (Fig. 4C).

According to the formula Risk score = 0.311 × MTHFD1 + (0.204) × PDGFB + (−0.277) × PIK3R3 + (−0.207) × CHKB, the risk score of each patient in TCGA-LUAD (training cohort) and Orient-11 study (validation cohort) was calculated. All the patients were divided into high-risk group and low-risk group based on the median value of risk score. Kaplan–Meier survival analysis demonstrated a significant difference in overall survival between two risk group, with the patients in high-risk group having poorer survival and higher risk of death for both training (*p* = 0.00023) (Fig. 4D) and validation cohort (*p* = 0.00039) (Fig. 4E). Additionally, we performed ROC curves and calculated AUCs of both training and validation cohorts. Results showed that the ROC curve had acceptable sensitivity and specificity, indicating the satisfactory prognostic and predictive power of the constructed choline metabolism-related signature (Additional file 3: Fig. S2C-D).

### Choline metabolism signature was associated with immunosuppressive tumor microenvironment

**Fig. 5:**
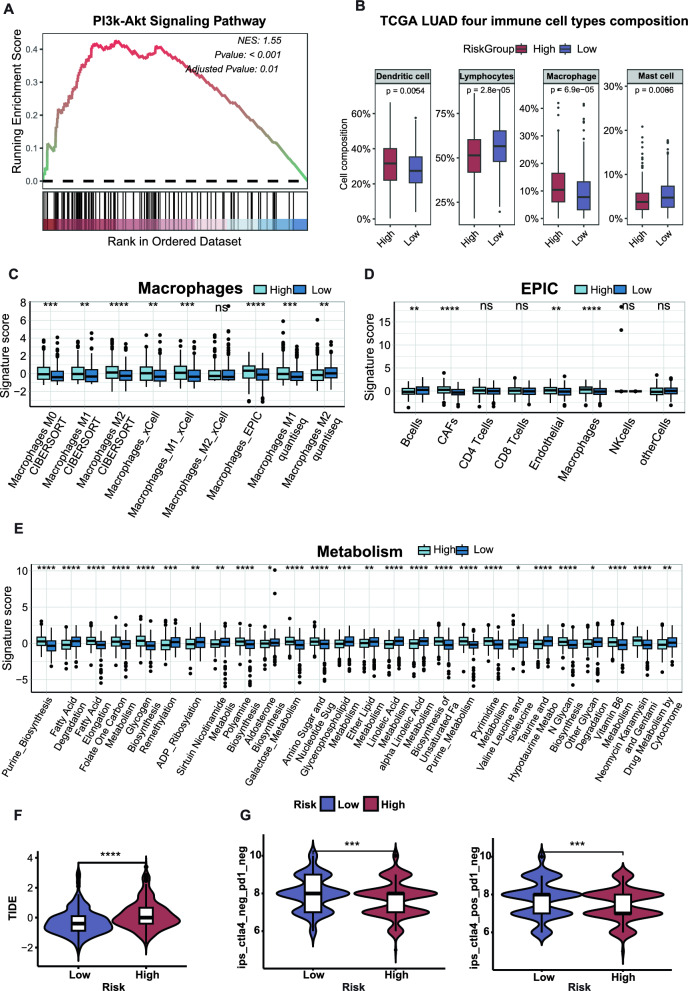
Immune cells infiltration, function enrichment analysis and immune evasion. **A** PI3k-Akt signaling pathway highly expressed in high-risk of choline metabolism. **B**–**C** Differential analysis of immune cell types composition (**B**) and subtypes of macrophages (**C**). **D** Estimating the Proportion of Immune and Cancer cells (EPIC) of different risk groups. **E** Comparison of metabolism-related gene sets in different risk groups. **F**–**G** Immune evasion and response. **F** TIDE score of the low- and high-risk groups of choline metabolism. **G** Sensitivity analysis of the two risk groups to immunotherapy. *ns* non-significant; *p < 0.05; **p < 0.01; ***p < 0.001; ****p < 0.0001

We performed the GSEA analysis used those upregulated and statistically significant DEGs in the high-risk group of the choline metabolism signature and results showed that multiple cancer-related pathways, including cell cycle, extracellular matrix (ECM) receptor interaction, cell motility, P53 and PI3k-Akt signaling were significantly enriched (Fig. 5A, Additional file 4: Fig. S3A). These results suggested that the high-risk of choline metabolism signature may have stronger ability of tumor cell growth, proliferation, invasion and metastasis of lung cancer. To further investigate the association between the choline metabolism signature and the immune microenvironment, we used the “IOBR” R package to calculate the scores of tumor microenvironment-related gene sets in the high-risk and low-risk groups of patients in TCGA-LUAD. We found that the gene sets related to lymphocytes, B cells and Mast cells were down-regulated in high-risk patients, whereas the gene sets related to macrophages, CAF cells and endothelial cells were significantly up-regulated (Fig. 5B–D). By comparing the score of metabolism-related gene sets, significant differences in multiple metabolism-related pathways were found between high-risk and low-risk group, suggesting the phenomenon of metabolic reprogramming in patients with high-risk (Fig. 5E). Furthermore, TIDE scores in high-risk group of choline metabolism signature were considerably greater than those in the low-risk group (Fig. 5F). Consistently, patients in the high-risk group of choline metabolism possessed a worse response rate to ICIs (ctla4_pos_pd1_neg, ctla4_neg_pd1_neg, *p* < 0.001, Fig. 5G), indicating that higher risk of choline metabolism may have stronger ability of immune evasion and immunotherapy resistance. Collectively, the above results suggested that patients in high-risk group were characterized by immunosuppressive tumor microenvironment.

### MTHFD1 was specifically expressed in tumor-associated macrophages (TAMs) and mediated immunosuppressive functions

**Fig. 6:**
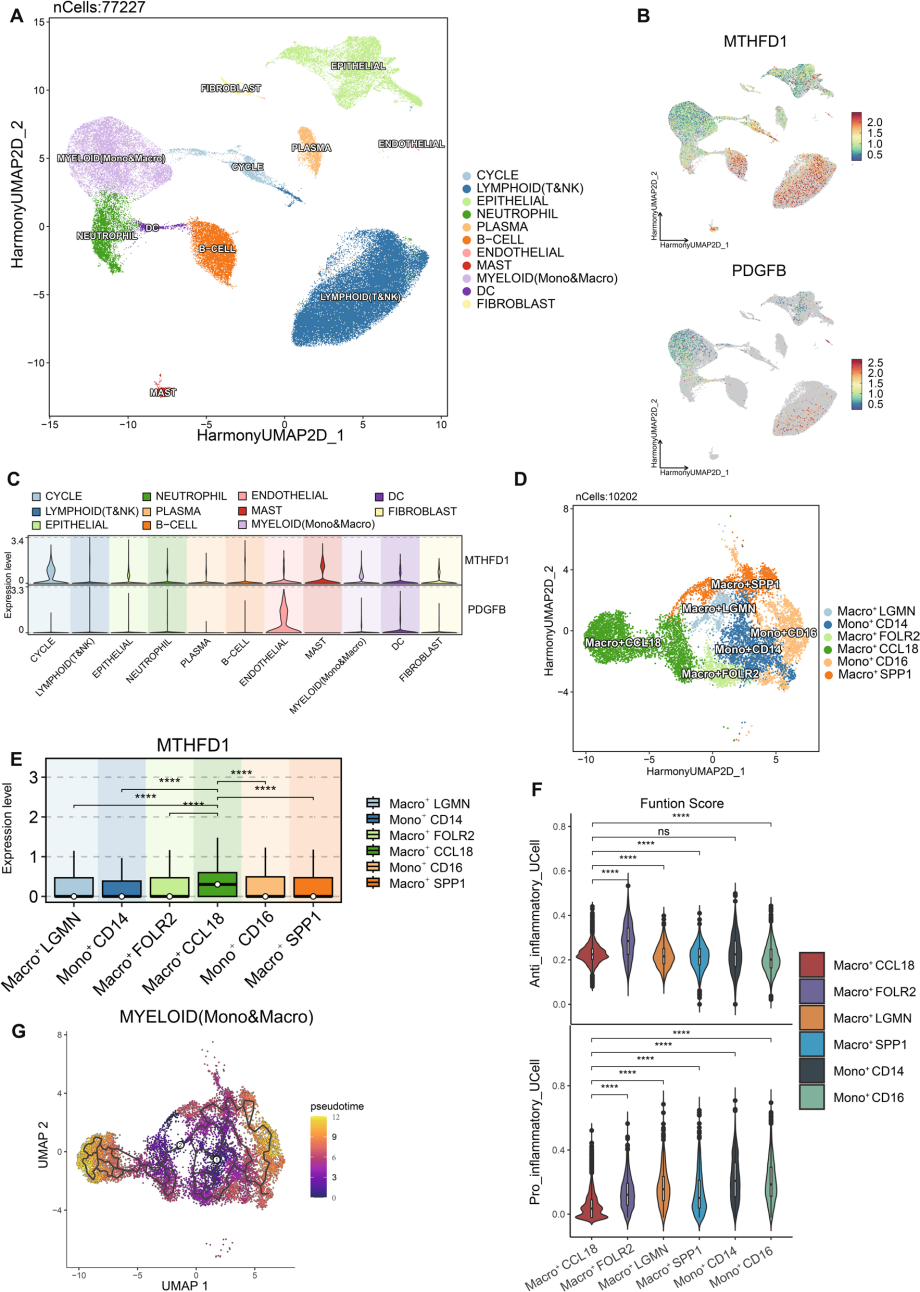
Single-cell sequencing data analysis. **A** Cell type annotation of 77,227 cells using uniform manifold approximation and projection (UMAP) plots. Feature plots (**B**) and grid violin plot (**C**) of single-cell dataset demonstrating expression of MTHFD1 and PDGFB across the 11 cell types. **D** UMAP showing grouping and annotation of myeloid cells. **E** Expression of MTHFD1 in different subgroups of myeloid cells. **F** Functional characteristics of 6 myeloid cell subsets calculated by Ucell score. **G** Pseudotime trajectory analysis of myeloid cells subsets. *ns* non-significant; ***p < 0.001; ****p < 0.0001

To further explore how choline metabolism mediates the immunosuppressive tumor microenvironment in NSCLC patients, single-cell sequencing data (GSE207422) were downloaded from GEO database, which contained fresh tumor samples from 3 pre-treatment and 12 post-treatment patients with NSCLC who received anti-PD-1 therapy combined with chemotherapy. After performing quality control on the samples, removing batch effects by Harmony analysis, we finally obtained 11 cell types by clustering in reduced dimensions and manual annotation (Fig. 6A). We found that MTHFD1 was mainly expressed in myeloid cells (monocytes & macrophages) and mast cells, while PDGFB was mainly expressed in endothelial cells (Fig. 6B, C). Our previous results demonstrated that multiple macrophage pathway scores were significantly up-regulated in high-risk patients, we further explored the expression of MTHFD1 in myeloid cells. After further grouping and annotation of myeloid cells, 6 subgroups of myeloid cells can be obtained (Fig. 6D). We found that MTHFD1 expression gradually increased as monocytes differentiate into macrophages and it was mainly expressed in CCL18^+^ macrophages (Fig. 6E), and meanwhile CCL18 expression was up-regulated in TCGA-LUAD patients with high-risk (Additional file 3: Fig. S2E), suggesting that MTHFD1 may be implicated in immunosuppressive macrophage differentiation of NSCLC. Ucell score was then used to calculate the functional characteristics of 6 myeloid cell subsets, and results showed that CCL18^+^ macrophage had both a higher anti-inflammatory related score and a lower pro-inflammatory related score (Fig. 6F), suggesting that CCL18^+^ macrophage belonged to immunosuppressive subsets. To infer the developmental trajectory of myeloid cells, monocle3 (v0.2.1) was used to perform pseudotime analysis and results indicated that the subset of CCL18^+^ macrophages belonged to the endpoint of differentiation (Fig. 6G).

To deepen the understanding of the connection between MTHFD1 and macrophages polarization, we stratified all TCGA-LUAD patients into low and high expression groups of MTHFD1 based on the median level. We observed that high expression of MTHFD1 was correlated with elevated level of M2-like macrophages, consistent with previous findings associating high-risk group of choline metabolism with increased M2-like macrophages (Additional file 4: Fig. S3B). Furthermore, GSEA showed the downregulation of M1-like macrophages-related gene sets in MTHFD1-high patient tumors compared with MTHFD1-low patient tumors (Additional file 4: Fig. S3C). Correlation analysis verified a positive association between MTHFD1 expression and CCL18^+^ immunosuppressive macrophages, collectively indicating that increased level of MTHFD1 may be associated with the polarization and immunosuppressive functions of macrophage in the tumor microenvironment (Additional file 4: Fig. S3D).

### Abnormal choline metabolism may affect endothelial cell proliferation by mediating the differentiation of macrophages

**Fig. 7:**
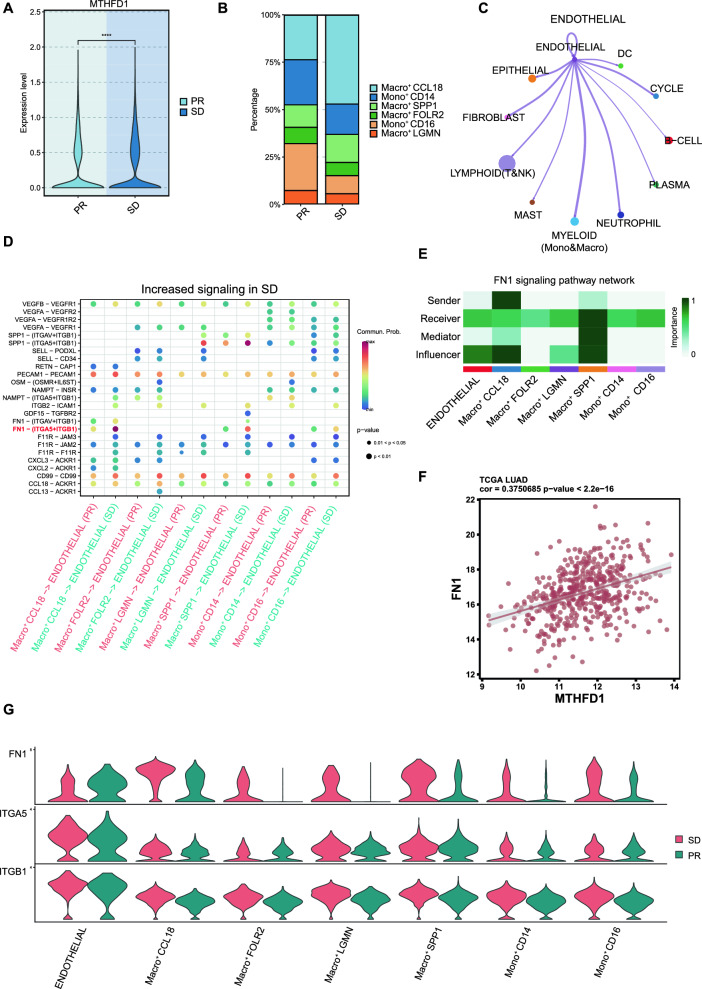
Cell–cell communication between endothelial cells and myeloid cells subsets. **A** Comparison of the expression level of MTHFD1 in partial-response (PR) group and stable-disease (SD) group. **B** Bar charts of 6 myeloid cell subsets proportions in PR and SD group. **C** Circle plot of the cell–cell communication between endothelial cells and myeloid cells subsets. **D** Bubble plot shows the ligand-receptor pairs contributing to the signaling from myeloid cells subsets to endothelial cells. **E** CellChat heat map showing the sender, receiver, mediator and influencer roles of endothelial cells and myeloid cells subsets for the FN1 signaling pathway. Color intensity shows the importance of the cluster contribution to each role. Dark green, high; white, low importance. **F** Correlation analysis between the expression level of FN1 and MHTFD1 in TCGA-LUAD cohort. **G** Violin plot showing the comparison of the expression distribution of FN1 signaling ligand and receptor genes in endothelial cells and myeloid cells subsets of PR and SD groups

Those post-treatment samples of single-cell sequencing data were further divided into two groups based on Response Evaluation Criteria in Solid Tumors Version (RECIST): partial-response group (PR; n = 8) and stable-disease group (SD; n = 4). We found that patients in the SD group had a higher expression of MTHFD1 (Fig. 7A), suggesting that higher expression of MTHFD1 may be associated with poor efficacy of immunotherapy. At the same time, by comparing the proportion of 6 myeloid cell subsets in PR and SD group, a significant difference was observed in the proportion of CCL18^+^ macrophages between two groups, with SD group exhibiting a higher proportion (Fig. 7B). These results above suggested that the expression of MTHFD1 was associated with the immunosuppressive functions of CCL18^+^ macrophages and may account for poorer immunotherapeutic effect.

PDGFB, another risk gene of choline metabolism in this study, was mainly expressed in endothelial cells as mentioned above (Fig. 6B, C). It was reported in previous research that higher expression of PDGFB in gastric cancer promoted angiogenesis in metastases via activation of the MAPK/ERK signaling pathway in endothelial cells [23]. Thus, it was reasonable to speculate that the immunosuppressive functions of macrophages in abnormal choline metabolism may be associated with the proliferation of endothelial cells to some extent. CellChat was next performed to identify the intercellular communication and results showed that endothelial cells had strong communication with myeloid cells (Fig. 7C), which was consistent with our previous findings that endothelial cell gene set scores were up-regulated in patients of high-risk. Furthermore, we next compared the differences in intercellular communication between endothelial cells and myeloid subsets of PR and SD group. Results showed that FN1- (ITGA5 + ITGB1) signaling pathway mediating the strongest communication probability from CCL18^+^ macrophages to endothelial cells in SD group (Fig. 7D), while this phenomenon was not significant in PR group. The hierarchy plot showed that CCL18^+^ macrophages were the most important sender in FN1 signaling pathway, endothelial cells were mainly the receiver, and both of them acquired high influencer scores (Fig. 7E), indicating a high capacity of the influencing information flow. By comparing the expression distribution of signaling genes involved in the inferred FN1 signaling network, a higher expression level of FN1 was observed in CCL18 macrophages of SD group compared with that of PR group (Fig. 7G). Finally, further correlation analysis indicated that there was a strong correlation between FN1 and MHTFD1 expression in TCGA-LUAD cohort (Fig. 7F). Previous studies have found that FN1 as well as ITGA5 could facilitate tumor angiogenesis and progression in cervical cancer [24, 25] and may be a prognostic risk factor in many types of cancer including Gliomas, gastric cancer, head and neck squamous cell carcinoma and NSCLC [26–30]. Above all, our results suggested that overexpression of MTHFD1 in abnormal choline metabolism contributes to the immune suppressive polarization in macrophages, further promoting the proliferation of endothelial cells. Together, these processes jointly shape the immunosuppressive microenvironment of NSCLC, ultimately mediating the occurrence of immunotherapy resistance.

## Discussion

Lung cancer is the leading cause of cancer and cancer-related death worldwide [31], imposing a tremendous health and financial burden. NSCLC accounts for 85% to 90% of lung cancer, and are mostly diagnosed at an advanced (locally advanced or metastatic disease) stage with a poor prognosis. Deregulation of metabolism has widely been recognized to play an important role in a variety of malignancies, due to its impact on tumor development, progression and treatment response [32, 33]. For instance, choline metabolism has been extensively studied in cancer research. Choline, a central metabolite in human metabolism, is an essential nutrient and methyl donor for epigenetic regulation [34, 35]. In normal physiological conditions, ChE is a key enzyme responsible for catalyzing the hydrolysis of ACh into choline and acetic acid [10], playing a crucial role in maintaining a balanced choline metabolism. Loss or defects in ChE expression or activity, as well as abnormal choline metabolism, could influence on the cell growth, motility and invasion capability of tumor cells. Understanding the mechanisms of choline metabolic changes in lung cancer may provide valuable insights for developing novel therapies to improve the survival outcomes of patients.

In this study, we confirmed that ChE level was significantly associated with survival outcomes of patients with advanced NSCLC undergoing immunotherapy. Patients with high ChE at baseline or an elevation of ChE after 2 cycles of immunotherapy had better clinical outcomes and response to immunotherapy. Moreover, univariate and multivariate Cox regression analysis showed that ChE, both at baseline and the early changes, were independent prognostic factors and had satisfactory prognostic accuracy and predictive value when included in the constructed nomogram. Therefore, monitoring the pretreatment as well as the dynamic changes in ChE level might contribute to powerful and effective biomarkers to identify the NSCLC patients who will probably most benefit from immunotherapy. Due to the convenience and cost-effectiveness of monitoring ChE, it makes it easier for clinical implementation.

To understand the mechanisms of choline metabolic changes, we further investigated the role of choline metabolism in lung cancer. Based on the TCGA-LUAD databases and the gene sets of choline metabolism-related genes, we obtained a signature of four choline metabolism-related prognostic genes, including two risk genes (MTHFD1, PDGFB) and two protection genes (PIK3R3, CHKB). TCGA-LUAD database was used as training cohort and orient-11 database was used as validation cohort. The overall survivals of patients in the high-risk group were significantly worse than those in the low-risk group. Also, the AUCs of the training cohort at 1 and 3 years were 0.634 and 0.618, suggesting that the choline metabolism-related signature has effective and reliable predictive power for the prognosis of LUAD patients.

The tumor microenvironment (TME) consists of cellular components (tumor cells, immune cells, fibroblasts, endothelial cells, and various stromal cells) and non-cellular components (extracellular matrix components, signaling molecules, cytokines, chemokines, and growth factors) [36, 37]. TME provides a supportive niche for tumor cells, facilitating their survival, proliferation, and evasion of immune responses [37]. However, the balance between pro- and anti-tumorigenic immune responses within TME is critical in determining tumor fate. Tumor-associated immune cells, such as TAMs, dendritic cells, and T cells, can possess either tumor-antagonizing or tumor-promoting functions depending on the context [38, 39]. Recently, the administrations of immunotherapy have brought a new era for cancer therapy, and it has been widely reported that the heterogeneity in TME shows a profound association with the tumor progression and responsiveness to immunotherapy [40]. In this study, we found that those gene sets related to macrophages, CAF cells and endothelial cells were significantly up-regulated in the high-risk group of choline metabolism. The proportion of anti-inflammatory was significantly elevated in high-risk group, which are reported be mainly involved in immunosuppression, tumorigenesis and metastasis [41]. Meanwhile, Endothelial cells could facilitate tumor angiogenesis and metastasis, create a physical barrier and secrete immunosuppressive [41, 42], which are key stromal components of the TME. Moreover, a significant phenomenon of metabolic programming and a higher score of TIDE were observed in high-risk group, indicating increased immune evasive potential and decreased responsiveness to immunotherapy. In summary, abnormal choline metabolism may contribute in the formation of immunosuppressive microenvironment, strengthen the capacity of immune escape and finally result in the poor effective of immunotherapy.

MTHFD1, methylenetetrahydrofolate dehydrogenase 1, plays a crucial role in choline metabolism by participating in the transfer and metabolism of one-carbon units [43]. MTHFD1 is reversibly catalyze the stepwise oxidation from 5,10-CH2-THF to 10-CHO-THF, and the conversion of 10-CHO-THF to THF [44], providing methyl donors for choline synthesis and maintaining normal choline levels. MTHFD1 is reported to be a potential oncogene in tumorigenesis, with a high expression in tumor cells. Excessive expression and activity of MTHFD1 may sustain high proliferative capacity of tumor cell, enhance invasive and metastatic capabilities, influence apoptosis and cell cycle regulation pathways, and be associated with poor prognosis and increased risk of recurrence in tumors [45–47]. In our study, we found that MTHFD1 is highly overexpressed in LUAD of TCGA database. Further single-cell analysis showed that MTHFD1 is specifically highly expressed in CCL18^+^ macrophages, which is consistent with our previous findings indicating enrichment of the macrophage pathway in high-risk patients. CCL18 is a chemokine secreted by tumor-associated macrophages that promotes a pro-tumor microenvironment by inducing a pro-tumor (M2-like) macrophage phenotype [48]. CCL18 is involved in tumor invasion, migration, epithelial-to-mesenchymal transition (EMT), and angiogenesis, ultimately contributing to cancer progression [49]. Also, it is reported that CCL18^+^ macrophages could activate NF-κB pathway in fibroblasts and induce the stemness and resistance of cancer cells [50].In this study, pseudotime analysis was performed to gain further insight into mechanisms and results indicated that MTHFD1 expression increases during monocyte-to-macrophage differentiation, suggesting its role in immunosuppressive macrophage differentiation in NSCLC. To validate the correlation between the MTHFD1 and macrophage polarization, we also stratified patients based on the median level of MTHFD1. High expression of MTHFD1 was observed with elevated level of M2-like macrophages, along with downregulation of M1-like macrophages-related gene sets. Correlation analysis also verified a positive association between MTHFD1 expression and CCL18^+^ macrophages. Our findings collectively suggested that MTHFD1 may contribute to the immunosuppressive functions of CCL18^+^ macrophages and potentially lead to a poorer immunotherapeutic response.

Previous studies have demonstrated that high expression of FN1 is associated with poorer survival outcomes and treatment efficacy in multiple cancers [51, 52]. FN1 also plays a significant role in the tumor microenvironment. It is closely related to tumor proliferation, invasion, EMT processes and immune infiltration levels [53]. Moreover, it has been reported that macrophages can drive resistance through the cytokine activin A, which induces the FN1-ITGA5-SRC signaling cascade [54]. In our study, the upregulation of the FN1 signaling pathway between CCL18^+^ macrophages and endothelial cells, particularly in the non-responder group, indicated their potential role in promoting endothelial cell proliferation and tumor angiogenesis in NSCLC patients. Strong correlations between FN1 and MTHFD1 expression were observed in both single-cell sequencing data and the TCGA cohort, further supporting their association in NSCLC. These findings provide insights into the role of MTHFD1 in NSCLC pathogenesis and highlight its potential as a therapeutic target.

Undeniably, this study has several limitations. Firstly, it is important to note that the retrospective design and the use of a moderate sample size from a single cancer institution may limit the generalizability of the findings regarding the association between ChE and clinical outcomes. Secondly, the utilization of research data (transcriptomic or single-cell datasets) from public databases such as TCGA, GEO, KEGG introduces potential limitations and incomplete information, emphasizing the need for validation with a larger sample size to ensure accurate and generalizable findings. Finally, gaining a comprehensive understanding of the specific molecular mechanisms involved in the choline metabolism-related signature in the pathogenesis of lung cancer requires additional molecular biology experiments.

## Conclusion

In conclusion, our study emphasizes the initial discovery of the prognostic value of ChE in advanced non-small cell lung cancer, highlighting its potential as a valuable and cost-effective marker to identify patients who are more likely to benefit from immunotherapy. Furthermore, this study developed a prognostic signature for lung adenocarcinoma based on choline metabolism-related genes, demonstrating its correlation and impact on the immunosuppressive microenvironment. Specifically, the study uncovers the overexpression of MTHFD1 in abnormal choline metabolism was intimately associated with TAMs, shedding light on its role in immunosuppressive macrophage differentiation and endothelial cell proliferation, thus providing valuable insights into the intricate workings of choline metabolism in NSCLC pathogenesis.

### Supplementary Information

Supplementary material 1: Figure S1. The flow chart of patients screening.

Supplementary material 2: Figure S2. (A)The calibration curve for predicting the 1-year, 2-year and 3-year OS of patients. (B) The decision curve analysis (DCA) curve for the constructed nomogram. (C-D) The prognostic accuracy of the nomogram was estimated by using ROC curves in the training cohort (C) and the validation cohort (D). (E) Comparison of CCL18 expression between high-risk and low-risk groups of choline-metabolism signature in TCGA-LUAD patients.

Supplementary material 3: Figure S3. (A) Pathway enrichment bubble plots from GSEA analysis. The bubble area corresponds to the GSEA normalized enrichment score (NES); the intensity of the color corresponds to the statistical significance of the enrichment. (B) Violin plot comprising the expression of M2-like macrophages in low/high level groups of MTHFD1. (C) GSEA analysis comparing the M1-like macrophages-up related gene sets between MTHFD1-high and MTHFD1-low patient tumors. (D) Correlation analysis between the expression level of MHTFD1 and CCL18 in TCGA-LUAD cohort. *p<0.05.

Supplementary material 4: Table S1. All the choline metabolism-related genes analyzed in this study.

Supplementary material 5: Table S2. Predictive factors for progression-free survival (PFS) by univariate and multivariate analysis.

## Data Availability

The datasets used and/ or analyzed during this study are available from the corresponding author on reasonable request.
