# Supplementary material for: Choline metabolism reprogramming mediates an immunosuppressive microenvironment in non-small cell lung cancer (NSCLC) by promoting tumor-associated macrophage functional polarization and endothelial cell proliferation
Source: J Transl Med. 2024 May 10;22:442. doi: 10.1186/s12967-024-05242-3 (PMC11084143; doi:10.1186/s12967-024-05242-3)
Supplement: Supplementary file 1 — Supplementary material 1: Figure S1. The flow chart of patients screening. [file 12967_2024_5242_MOESM1_ESM.pdf]

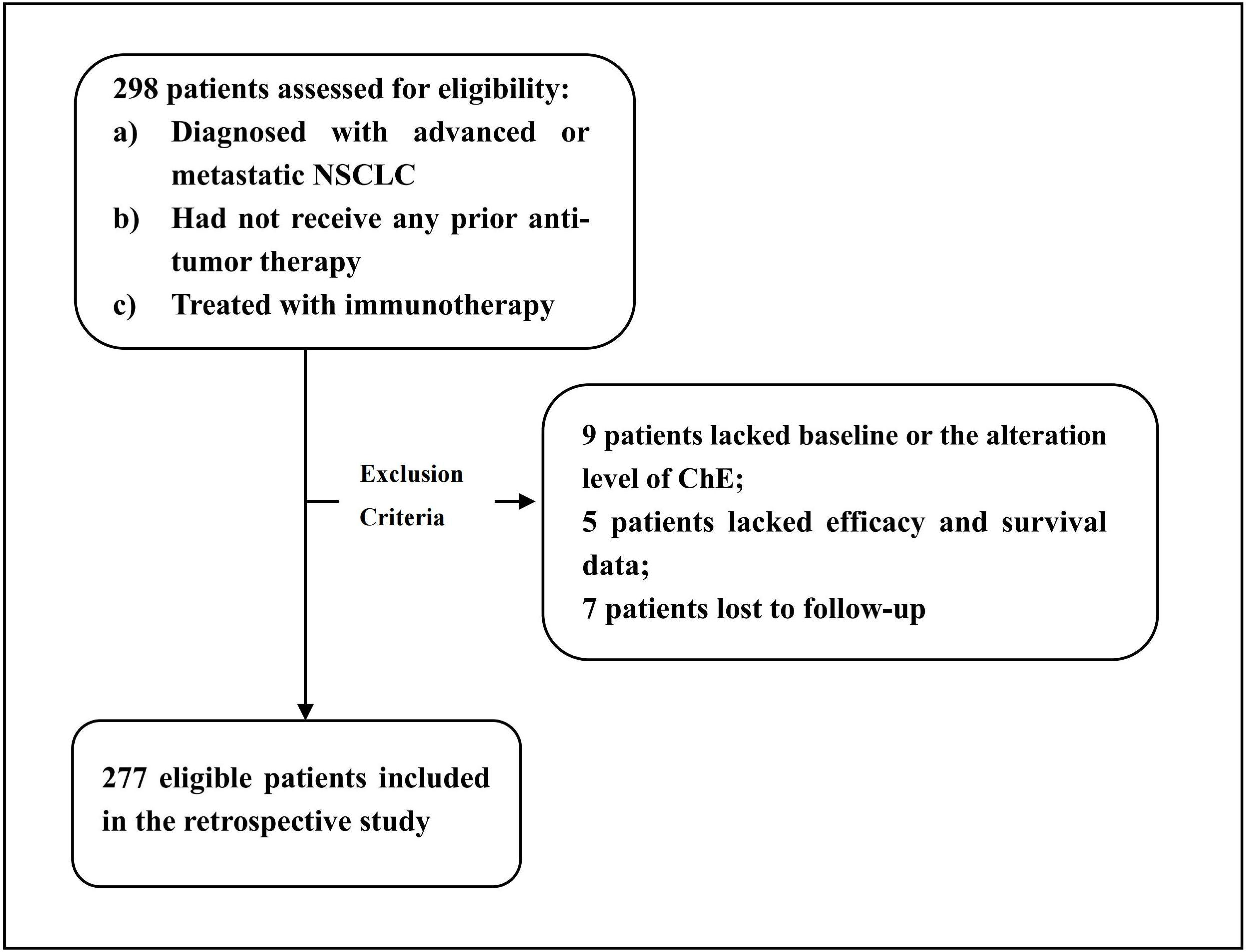
298 patients assessed for eligibility:
a) Diagnosed with advanced or metastatic NSCLC
b) Had not receive any prior anti-tumor therapy
c) Treated with immunotherapy
Exclusion Criteria
9 patients lacked baseline or the alteration level of ChE;
5 patients lacked efficacy and survival data;
7 patients lost to follow-up
277 eligible patients included in the retrospective study
