# Supplementary figures and images for: Choline metabolism reprogramming mediates an immunosuppressive microenvironment in non-small cell lung cancer (NSCLC) by promoting tumor-associated macrophage functional polarization and endothelial cell proliferation
Source: J Transl Med. 2024 May 10;22:442. doi: 10.1186/s12967-024-05242-3 (PMC11084143; doi:10.1186/s12967-024-05242-3)

A

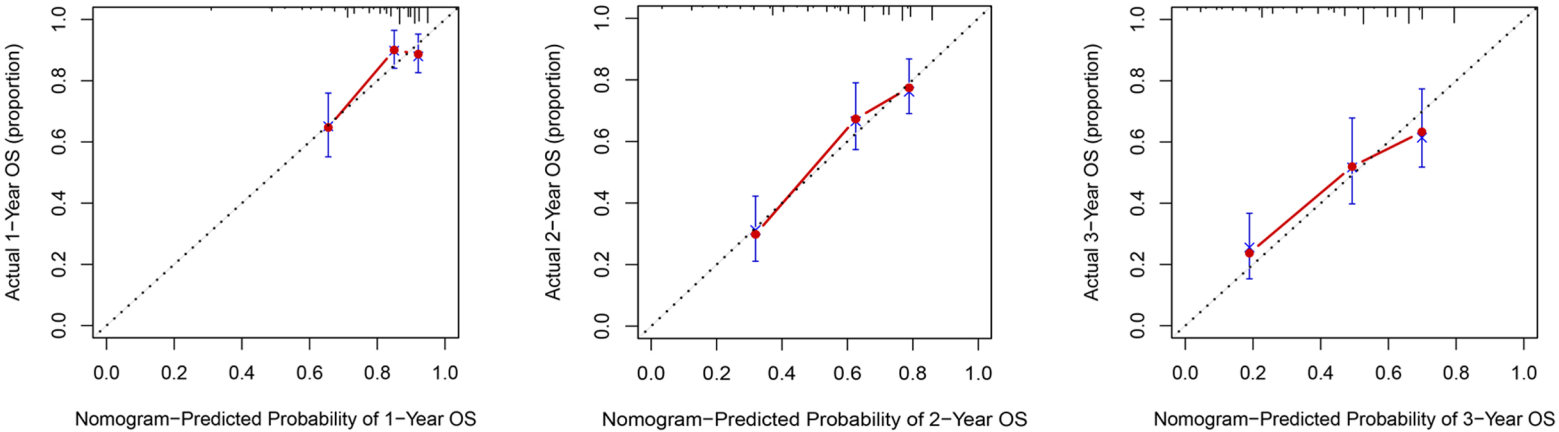

B

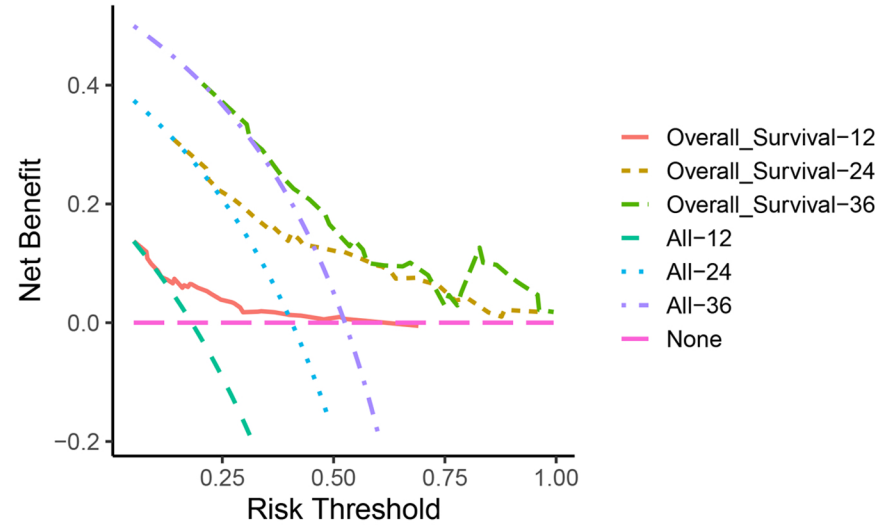

E

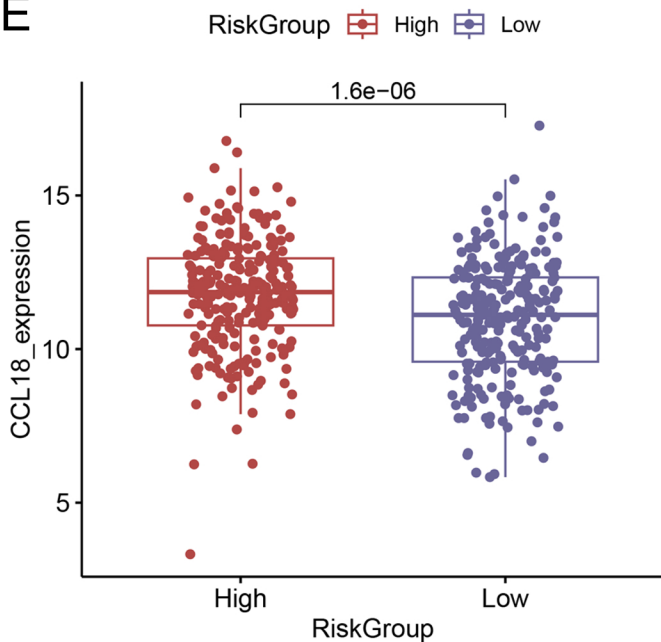

C

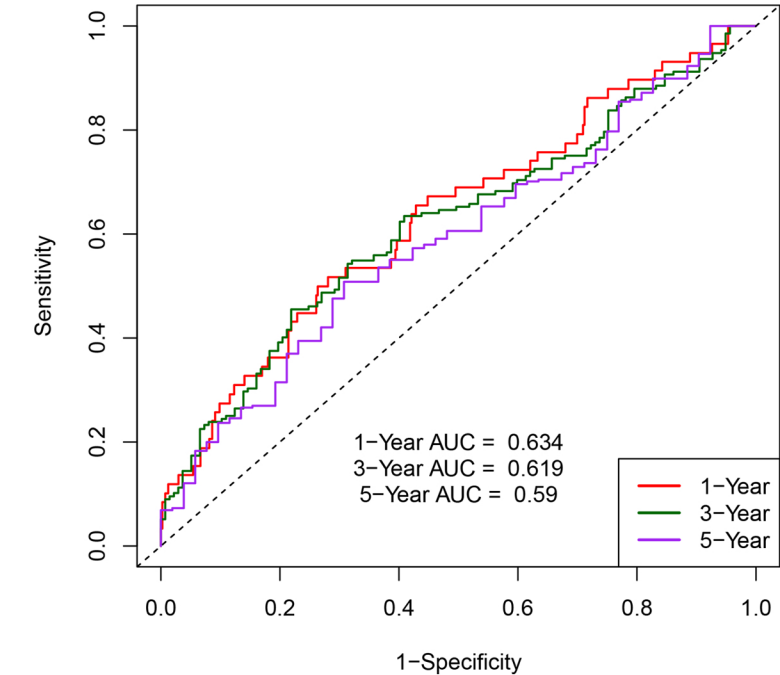

D

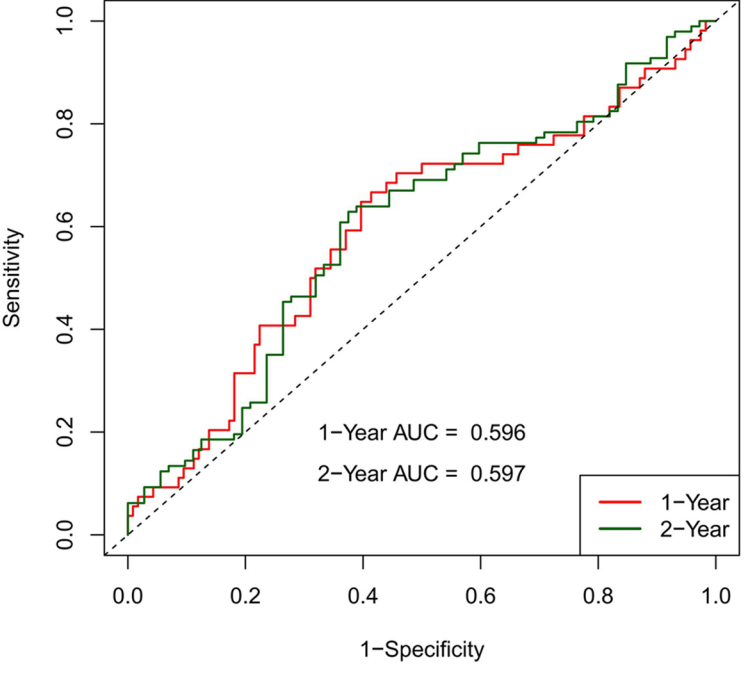

Supplement: Supplementary file 2 — Supplementary material 2: Figure S2. (A)The calibration curve for predicting the 1-year, 2-year and 3-year OS of patients. (B) The decision curve analysis (DCA) curve for the constructed nomogram. (C-D) The prognostic accuracy of the nomogram was estimated by using ROC curves in the training cohort (C) and the validation cohort (D). (E) Comparison of CCL18 expression between high-risk and low-risk groups of choline-metabolism signature in TCGA-LUAD patients. [file 12967_2024_5242_MOESM2_ESM.pdf]

A

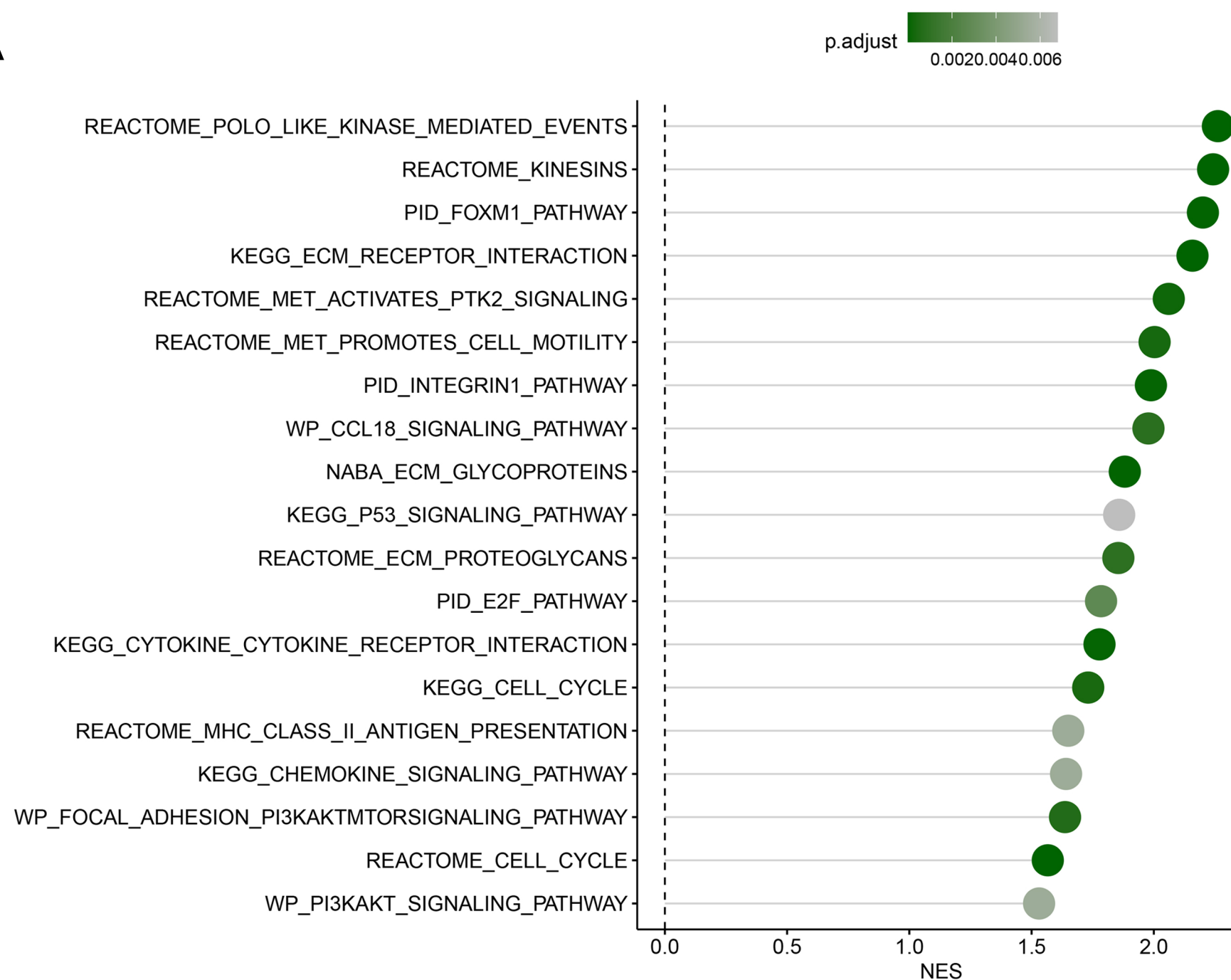

B

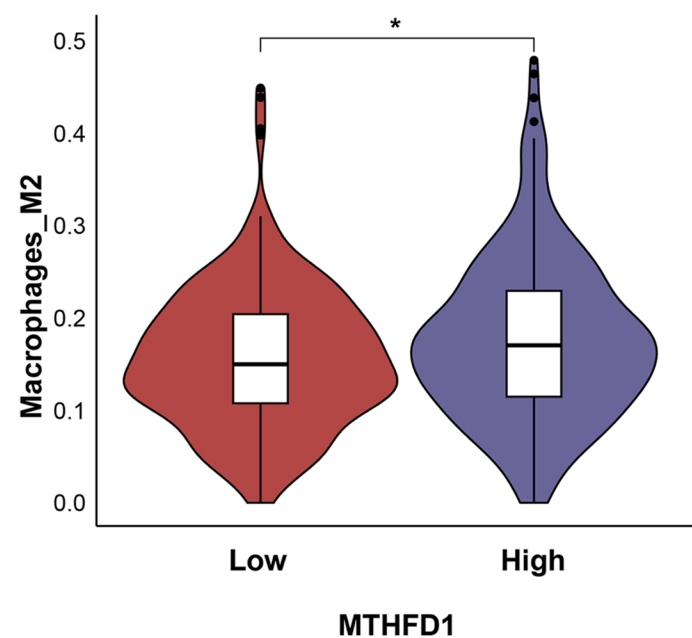

C

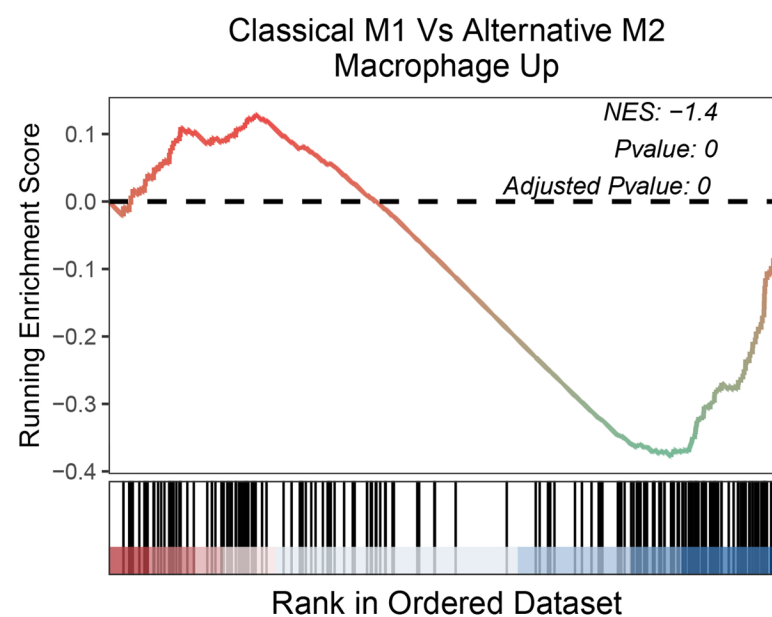

D

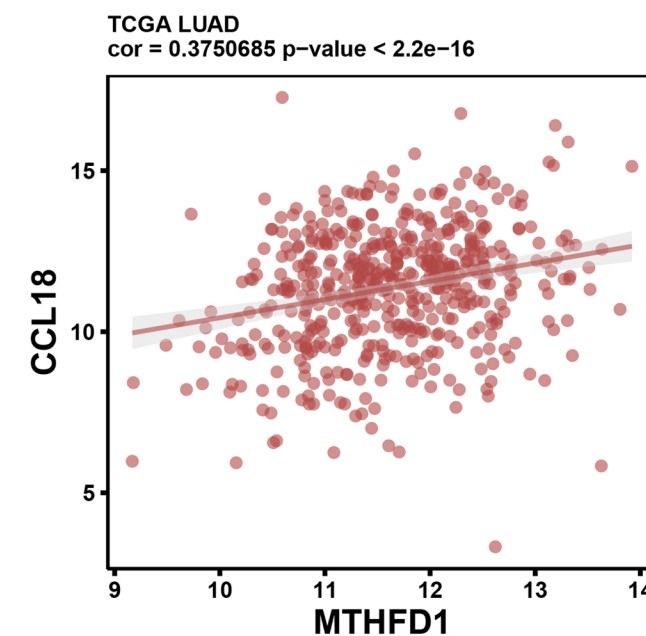

Supplement: Supplementary file 3 — Supplementary material 3: Figure S3. (A) Pathway enrichment bubble plots from GSEA analysis. The bubble area corresponds to the GSEA normalized enrichment score (NES); the intensity of the color corresponds to the statistical significance of the enrichment. (B) Violin plot comprising the expression of M2-like macrophages in low/high level groups of MTHFD1. (C) GSEA analysis comparing the M1-like macrophages-up related gene sets between MTHFD1-high and MTHFD1-low patient tumors. (D) Correlation analysis between the expression level of MHTFD1 and CCL18 in TCGA-LUAD cohort. *p<0.05. [file 12967_2024_5242_MOESM3_ESM.pdf]
