# Supplementary material for: Choline metabolism reprogramming mediates an immunosuppressive microenvironment in non-small cell lung cancer (NSCLC) by promoting tumor-associated macrophage functional polarization and endothelial cell proliferation
Source: J Transl Med. 2024 May 10;22:442. doi: 10.1186/s12967-024-05242-3 (PMC11084143; doi:10.1186/s12967-024-05242-3)
Supplement: Supplementary file 5 — Supplementary material 5: Table S2. Predictive factors for progression-free survival (PFS) by univariate and multivariate analysis. [file 12967_2024_5242_MOESM5_ESM.docx]

**Table S2 Predictive factors for PFS by univariate and multivariate analysis**

|  |  | **Univariate analyses** | | **Multivariate analyses** | |
| --- | --- | --- | --- | --- | --- |
|  |  | **HR (95%CI) *p* value^Φ^** | | **HR (95%CI) *p* value^Φ^** | |
| Gender | male vs. female | 1.02(0.72-1.44) | 0.924 |  |  |
| Age | <62 vs. ≥62 | 0.89(0.67-1.18) | 0.427 |  |  |
| ECOG-PS | 1-2 vs. 0 | 1.20(0.90-1.59) | 0.221 |  |  |
| Smoking status | Yes vs. No | 1.06(0.79-1.42) | 0.715 |  |  |
| Liver metastasis | Yes vs. No | 2.02(1.39-2.94) | **<0.001** | 1.90(1.30-2.78) | **0.001** |
| Brain metastasis | Yes vs. No | 0.98(0.73-1.32) | 0.895 |  |  |
| Bone metastasis | Yes vs. No | 1.50(1.12-2.01) | **0.006** | 1.47(1.10-1.98) | **0.009** |
| Baseline ChE, U/L | ≥7611 vs. <7611 | 0.72(0.54-0.96) | **0.027** | 0.64(0.48-0.87) | **0.004** |
| ChE alteration, U/L | ≥0 vs. <0 | 0.56(0.42-0.75) | **<0.001** | 0.51(0.37-0.68) | **<0.001** |
| LDH, U/L | ≥210 vs. <210 | 0.95(0.72-1.26) | 0.731 |  |  |
| ALB, g/L | ≥42 vs. <42 | 0.91(0.68-1.21) | 0.500 |  |  |
| NLR | ≥3.18 vs. <3.18 | 1.00(0.75-1.33) | 0.994 |  |  |
| CRP, mg/L | ≥11 vs. <11 | 1.14(0.86-1.52) | 0.363 |  |  |
| SAA, mg/L | ≥20 vs. <20 | 1.24(0.93-1.64) | 0.144 |  |  |

Abbreviations: PFS, progression-free survival; ECOG-PS, Eastern Cooperative Oncology Group performance status; ChE, Cholinesterase; LDH, lactate dehydrogenase;

ALB, serum albumin; NLR, neutrophil-to-lymphocyte ratio; CRP, C-reactive protein; SAA, serum amyloid A;

HR Hazard ratio, CI Confidence interval. **^Φ^**Values in boldface indicate *p* values <0.05.
